# Effects of Music and White Noise Exposure on the Gut Microbiota, Oxidative Stress, and Immune-Related Gene Expression of Mice

**DOI:** 10.3390/microorganisms11092272

**Published:** 2023-09-10

**Authors:** Zhenyu Zhang, Yinqiang Wu, Shizheng Zhou, Pengcheng Fu, Hong Yan

**Affiliations:** State Key Laboratory of Marine Resource Utilization in South China Sea, Hainan University, Haikou 570228, China

**Keywords:** microbiota, oxidative stress, music, white noise, qPCR

## Abstract

The microbiota in gastrointestinal tracts is recognized to play a pivotal role in the health of their hosts. Music and noise are prevalent environmental factors in human society and animal production and are reported to impact their welfare and physiological conditions; however, the information on the relationship between the microbiota, physiological status, and sound is limited. This study investigated the impact of music and white noise exposure in mice through 16s rRNA gene sequencing, enzyme assay, and qPCR. The results demonstrate that white noise induced oxidative stress in animals by decreasing serum SOD and GSH-PX activity while increasing LDH activity and MDA levels (*p* < 0.05). Conversely, no oxidative stress was observed in the music treatment group. The relative gene expression of IFN-γ and IL-1β decreased in the white noise group compared to the music and control groups. The 16s rRNA gene amplicon sequencing revealed that *Bacteroidetes*, *Firmicutes*, *Verrucomicrobia*, and *Proteobacteria* were dominant among all the groups. Furthermore, the proportion of *Firmicutes* increased in the music treatment group but decreased in the white noise treatment group compared to the control group. In conclusion, white noise has detrimental impacts on the gut microbiota, antioxidant activity, and immunity of mice, while music is potentially beneficial.

## 1. Introduction

Music and noise are important environmental factors impacting human and animal health [1]. In the early 1950s, Gheerbrant made an important discovery regarding the soothing effects of Mozart’s “Sonata For Two Pianos in D Major, K.448” (Mozart K.448, K.448). Thereafter, the music piece was implemented as a representative piece of classical music [2,3] and sparked numerous subsequent studies exploring the influence of Mozart’s compositions [3,4]. Lin et al. investigated the long-term effects of Mozart K.448 on children with refractory epilepsy and found an average seizure reduction of 53.6 ± 62.0% [5]. The impact of music on improving animal health has also been reported in recent years. Barcellos et al. reported that musical intervention could decrease peripheral levels of pro-inflammatory cytokines and relieve anxiety among zebrafish (*Danio rerio*) [6]. Music has been widely used in agricultural practices, such as rainbow trout farming in recirculating water systems (RWS) [7] and milk production in automatic milking systems (AMS) for dairy cows [8]. It has also been implemented in animals kept in intensive production systems to reduce stress and improve their well-being [9]. Backus et al. have found that music could lower respiratory frequency values and improve the animal behavior of sows housed in collective stalls [10]. Li et al. have studied the effect of Mozart K.448 (60–70 dB) on the horizontal immunity of growing pigs and found that long-term music stimulus (60 d) enhanced their immunity through increasing immunoglobulin G (IgG), interleukin-2 (IL-2), and interferon-gamma (IFN-γ) levels (*p* < 0.05) [2].

Noise is a prevalent issue in animal health. Modern large-scale and intensive animal production generates noise through high-density housing, ventilation systems, feeding machines, automatic feces removal systems, etc. [11]. The decibel (dB) is a widely implemented unit to measure the intensity of sound, especially noise [12]. The noise level measured in dB for different processes of animal production has been widely reported. For animal housing, the noise level measured in a cattle barn ranged from 75 to 90 dB [13]; the noise levels measured in mechanically ventilated pig farms are around 73 dB [14]; the noise levels for laying hen farms range from 50 to 90 dB [15]. Even for laboratory animal facilities, noise levels exceeding 75 dB have also been reported [16]. For the animal in transportation and waiting for slaughter at the abattoir, the noise level ranges from 85 to 97 dB [14]. High-pitched and intermittent noises are generally considered irritating and can cause stress among animals. Broucek et al. found that tractor engine noise (97 dB) significantly increased blood glucose concentration, leukocyte counts, and hemoglobin levels [17]. Li et al. discovered that long-term noise exposure (80–85 dB) reduced the level of IgG in growing pigs (*p* < 0.05), indicating a detrimental effect of noise on animals [2]. Furthermore, recent research studies have indicated that noise from marine anthropogenic activities (deep-sea mining, seismic airgun, shipping, etc.) can negatively affect marine species ranging from plankton to whales, causing acoustic masking, cochlear damage, animal behavior disruption, physiological stress, hampered population recruitment for feeding and reproduction, and subsequently affecting the health of marine ecosystems and the yields of marine fisheries [18,19,20].

Oxidative stress refers to an imbalance between the accumulation of reactive oxygen species (ROS) and the antioxidant mechanisms in cells and tissues [21]. The key enzymes involved in the antioxidant mechanism include superoxide dismutase (SOD), glutathione peroxidase (GSH-Px), thioredoxin (Trx), and paraoxonase, which play a crucial role in maintaining redox homeostasis [22]. Failure of the antioxidant mechanism causes an increase in extracellular lactate dehydrogenase (LDH) [23], which facilitates the lipid peroxidation process. Accumulation of lipid peroxidation could damage cell integrity and cause a rise in malondialdehyde (MDA) concentration, which has been determined by an in vitro method [24]. Previous studies have linked sound exposure to oxidative stress in both humans and animals [25]. Manikandan et al. reported that exposing rats to white noise (100 dB) for 30 days (4 h/d) led to upregulation of SOD, increased lipid peroxidation in the brain (indicating cognitive impairment), and higher plasma corticosterone levels (indicating lower glutathione concentrations) [26]. Demirel et al. investigated the effect of noise on male Sprague-Dawley rats (20 days, 4 h/d) and found a significant increase in their MDA levels and GSH-Px activities [27].

The gut microbiota refers to the diverse community of microorganisms residing in the gastrointestinal tract, which plays a crucial role in maintaining the physiological homeostasis of the hosts. Approximately 95% of microbes dwell in the gut and influences the digestion, nutrition, growth, and immunity of the host [28]. The relationship between the gut microbiota (GM) and the host is reciprocal, forming the microbiota–gut–brain axis, in which the GM produces substances to facilitate the metabolism of the host, and the host’s physiological status has an impact on the composition of microbiota [29]. There is a growing interest in understanding how music or noise might influence gut microbiota. Preliminary research suggests that music, particularly soothing or relaxing music, may have indirect effects on gut microbiota by modulating stress levels and promoting a positive emotional state. Stress has been shown to influence the composition and diversity of gut microbiota, and music has been recognized for its potential to reduce stress and induce relaxation [29]. On the other hand, some studies have suggested that noise-induced stress can alter gut microbiota by promoting the growth of certain bacterial species or reducing microbial diversity. For example, animal studies have demonstrated that noise exposure can lead to changes in the gut microbial community, including shifts in the abundance of certain bacteria. Cui et al. found that chronic noise exposure changed the composition of the gut microbiota and negatively affected host immune responses and glucose metabolism, and even induced brain-related diseases, such as Alzheimer’s disease (AD) [30,31].

While the effects of music and noise on various aspects of human beings and/or animals, such as emotions, cognition, and physiological responses, have been studied for decades, few of them have explored the effects of both music and noise simultaneously. In essence, this study aimed to corroborate the effects of music or white noise on the composition of the microbial communities in the gut, and on the oxidative status and immunity of the mice to establish a possible relationship between them. Our findings could potentially be extended to the related human and animal research fields to provide initial insight into the impact of sounds on the microbiota–gut–brain axis.

## 2. Materials and Methods

### 2.1. Animal Model and Experimental Design

Male SPF C57BL/6J mice (six weeks old, weighing 18–20 g) were obtained from Hunan SJA 216 Laboratory Animal Co., Ltd. (Changsha, China). The mice were housed at a temperature of 23 ± 2 °C under a 12 h light/dark cycle (lights on at 8:00 a.m.), with a relative humidity of 60 ± 5%. The temperature and humidity in the room were maintained by a constant temperature and humidity air conditioning system (Gree Electric Appliances Inc., Zhuhai, China) and verified using a thermohygrometer (L-95-2, Shenzhen Haixu Instrument Co., Ltd., Shenzhen, China). They were provided ad libitum access to purified water and standard full-value chow (Hunan SJA Laboratory Animal Co., Ltd., Changsha, China). The animal husbandry followed the principles of laboratory animal care outlined by the China Animal Health and Epidemiology Center (www.cahec.cn, accessed on 8 May 2021). The animal experiments were reviewed and approved by the Hainan University Institutional Animal Welfare and Ethical Committee (HNUAUCC-2023-00169).

After a week-long adaptation period, the mice were randomly assigned to plastic cages (6 mice per cage) and divided into three groups (*n* = 7), which were marked as group A (Ga), group B (Gb), and group C (Gc). The mice in Ga were subjected to 3 h of music (Mozart K.448, Figure S1A). The mice in Gb were subjected to 3 h of white noise (Figure S1B,C). All the sound treatments were performed from 9:00 to 10:30 and 15:00 to 16:30, respectively. The sound loudness was maintained at 75–85 db and regularly monitored by a decibel meter (M-27 Dosimeter). Gc was selected as the control group with no sound treatment. The plastic cages for each group were isolated from one another and placed at a distance to minimize interference between the groups.

The experiment was extended for five weeks, and the body weight (BW) was measured weekly. At the end of the experiment, the mice were sacrificed by cervical dislocation and the mice were necropsied. Blood samples were collected by retro-orbital venous plexus under general anesthesia and kept at room temperature for 20 min, then the samples were centrifuged at 4 °C, 4000× *g*, for 10 min. The supernatant serum was extracted and stored at −80 °C for subsequent analysis. Fresh fecal samples were collected from the large intestine and stored at −80 °C for microbiota analysis.

### 2.2. Measurement of Serum Oxidative Stress Indicators

Malondialdehyde (MDA) [32], superoxide dismutase (SOD) [33], lactate dehydrogenase (LDH) [34], and glutathione peroxidase (GSH-Px) [35] in serum were quantitatively analyzed and calculated by the respective assay kit (Nanjing Jiancheng Chemical Industrial Co., Ltd., Nanjing, China) following the manufacturer’s instruction manual.

### 2.3. Quantitative RT-PCR

The total RNA was extracted from the samples by TRIzol (Thermo Fisher Scientific Inc., Waltham, MA, USA) according to the manufacturer’s instructions. The quantity of total RNA was determined by Nanodrop 2000 spectrophotometers (NanoDrop Technologies, LLC, Wilmington, NC, USA). Approximately 200 ng/μL total RNA for each sample was reversely transcribed with HiScript III All-in-one RT SuperMix Perfect for qPCR Kit (Vazyme, Nanjing, China). A real-time qPCR was conducted using ChamQ Universal SYBR qPCR Master Mix kit (Vazyme, Nanjing, China) and the reaction mixture was loaded into the ABI QuantStudio™ 6 Flex Real-time PCR System (ABI, Los Angeles, CA, USA). The target gene expression was normalized against the endogenous gene GADPH and calculated as a relative fold change to the control group with the 2^−∆∆Ct^ method [36]. The detailed targeted gene and primers are shown in Table S2.

### 2.4. 16s rRNA Gene Extraction and Sequencing

The microbial genomic DNA of the fecal samples from the large intestine was extracted by QIAamp DNA Stool Mini Kit (Qiagen, Valencia, CA, USA) according to the manufacturer’s protocol. A Qubit 2.0 fluorometer (Life Technology, Carlsbad, CA, USA) was used to determine the concentration and purity of each DNA extract. A polymerase chain reaction (PCR) amplification of the 16s rRNA gene V3/V4 region of the extracted DNA was performed with primers 338F and 806R. The PCR products were loaded onto agarose gel electrophoresis and the targeted sequence was purified with a Gel Extraction Kit (Qiagen, USA). The purified PCR was used to generate sequencing libraries and was sequenced with Illumina TruSeq (Illumina, San Diego, CA, USA).

### 2.5. Bioinformatics and Statistical Analysis

For the 16s rRNA gene sequencing data, the sequence quality was determined and samples with >150 sequences were further processed using Quantitative Insights into Microbial Ecology (QIIME) (version 1.8.0) [37]. The 16s rRNA gene sequences were assigned to operational taxonomic units (OTUs), which were compared against the SILVA database [38] at thresholds of 97% sequence similarity using UCLUST [39]. Representative sequences from each cluster were aligned with the PyNAST alignment tool [37]. The rarefaction curves [40], the Chao I [41], the Simpson index [42], and the Shannon index [43] were calculated using QIIME. Multiple alignments were performed using MUSCLE [44] to analyze the similarities among the samples. A phylogenetic tree and a principal coordinate analysis (PCoA) were performed using weighted UniFrac distances to find clusters of similar groups in the samples [45]. A Metastats analysis was performed to analyze the differential taxa between the groups (https://cbcb.umd.edu/software/metastats, accessed on 8 May 2021). A functional prediction based on the 16s rRNA gene sequencing data was performed using Tax4Fun [46]. The differences in the metabolic pathways among the groups were visualized by iPath3 [47]. The physiological data are presented as mean ± SEM. The data were analyzed using IBM SPSS Statistics for Windows, version 26 (IBMCorp., Armonk, NY, USA). The statistical differences among the groups were evaluated using the one-way analysis of variance (ANOVA) and the multivariate analysis of variance (MANOVA). A *p*-value < 0.05 was considered significant. OriginPro 2021b (OriginLab, Northampton, MA, USA) was employed for data visualization, and different lower-case letters above the error bars indicate a significant difference among the groups.

## 3. Results

### 3.1. Animal Weight Change and Behavior

**Figure 1:**
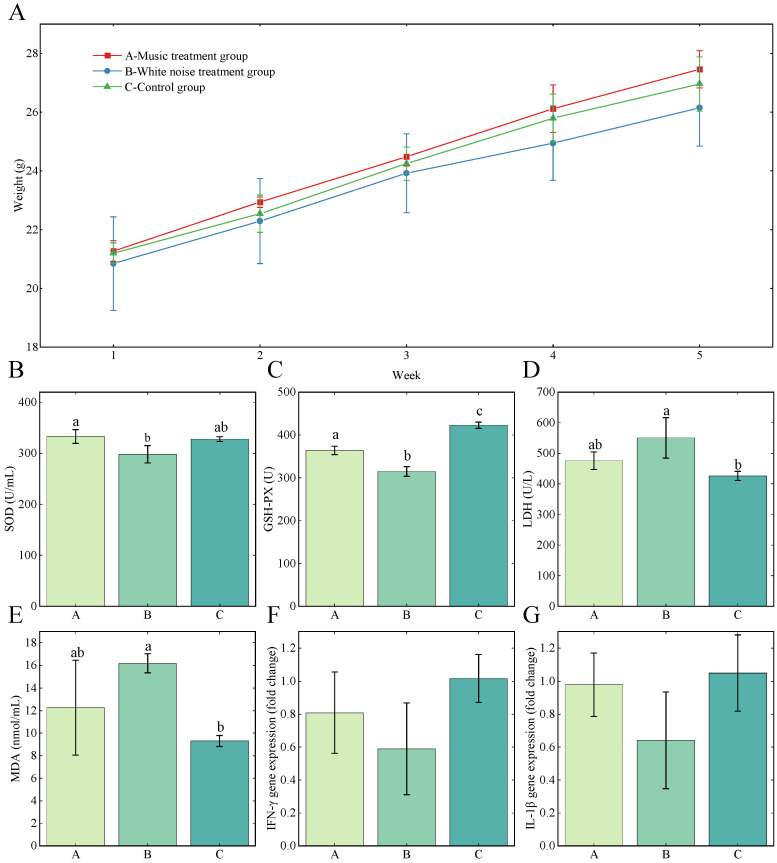
Weight (**A**), oxidation status indicators (SOD, GSH-Px, LDH, and MDA, (**B**–**E**)), and gene expression of IFN-γ and IL-1β (**F**,**G**). All the parameters are presented as mean ± SEM, and different lower-case letters above the error bars indicate a significant difference among different groups (*p* < 0.05).

The mice’s BW change is shown in Figure 1A. All the groups gained weight. It was seen that the BW of Gc (control group) was higher than Gb (white noise treatment group) and lower than Ga (music treatment group) throughout the 28 days of the experiment. The weight growth rate in Gb decreased after two weeks of white noise treatment. During the experiment period, the animals’ behavior was observed, and the mice with sound intervention (Ga and Gb) were more active than the control group.

### 3.2. Serum Oxidation Status Indicators Levels

The redox enzymes in serum, SOD, GSH-Px, LDH, and MDA are common oxidation status indicators (Figure 1B–F). Ga had the highest SOD level (332.93 ± 13.42 U/mL) compared with Gb (298.34 ± 17.19 U/mL, *p* < 0.05) and Gc (328.09 ± 4.67 U/mL). There was no significant difference between Gb and Gc regarding the SOD level. A significant difference in GSH-Px between the Gc (423 ± 7.34 U), Ga (364 ± 9.79 U), and Gb (315 ± 11.35 U) was observed (*p* < 0.05). For LDH, Gb (550.87 ± 66.41 U/L) had a significant difference compared with Gc (426.19 ± 14.90 U/L, *p* < 0.05) but had no significant difference with Ga (475.88 ± 28.53 U/L), and no significant difference was observed between Ga and Gc. The MDA concentration followed the same pattern as the LDH and had the highest MDA level at 16.18 ± 0.85 nmol/mL. The MDA for Ga and Gc were 12.25 ± 4.18 nmol/mL and 9.31 ± 0.49 nmol/mL, respectively.

### 3.3. Relative Expression Level of IFN-γ and IL-1β

Figure 1F,G shows the relative expression level of IFN-γ and IL-1β. Although there are no significant differences between the music treatment group (Ga), white noise treatment group (Gb), and the control group (Gc), the relative expression of IFN-γ and IL-1β were consistently lower in the white noise treatment group and approximately equal between the music treatment group and the control group.

### 3.4. 16s rRNA Gene Sequencing Data

**Figure 2:**
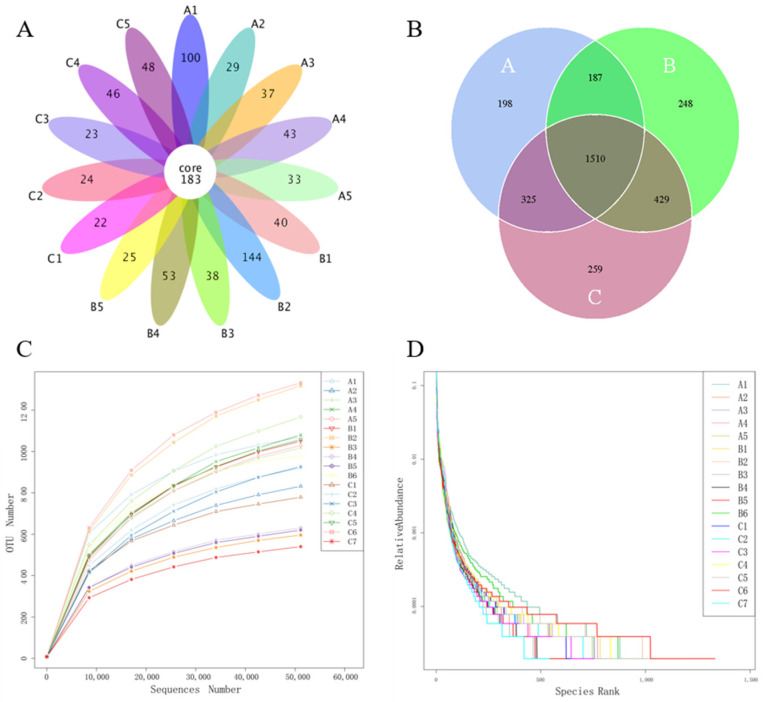
OTUs and sequencing data quality analysis. The flower plot shows the unique and common OTUs between the samples (**A**). The Venn plot shows the unique and shared OTUs between each group (**B**). The rarefaction curve (**C**) and the relative abundance curve (**D**) reflect the quality of the sequencing data.

The 16s rRNA gene sequencing resulted in a total of 1,591,970 primitive reads and 1,565,616 raw reads after sequence assembly. Subsequently, the low-quality reads from the raw reads were filtered through data denoising and chimera screening to obtain 1,155,869 effective reads, in which the music treatment group contains 318,541 reads, the white noise treatment group contains 386,833 reads, and the control group contains 450,495 reads (Table S1). To study the species composition of each sample, the effective tags of all the samples were clustered with 97% identity to obtain 3156 operational taxonomic units (OTUs). The core OTUs and unique OTUs for each sample are shown in Figure 2A. Figure 2B shows the common and different OTUs, among which the music treatment group (Ga) had 198 unique OTUs, the white noise treatment group (Gb) had 248 unique OTUs, and the control group (Gc) had 259 unique OTUs. A total of 187, 325, and 429 common OTUs were observed between Ga and Gb, Ga and Gc, and Gb and Gc, respectively. A total of 1510 common OTUs were observed between Ga, Gb, and Gc. In addition to the increasing sequence numbers, the rarefaction curve (Figure 2C) shows that the OTUs number for every sample plateaued and leveled out, indicating an adequate sequencing depth. The relative abundance curve (Figure 2D) decreased gradually and had a wide span on the x-axis, showing an even distribution of the species.

### 3.5. Microbial Diversity and Distribution

**Figure 3:**
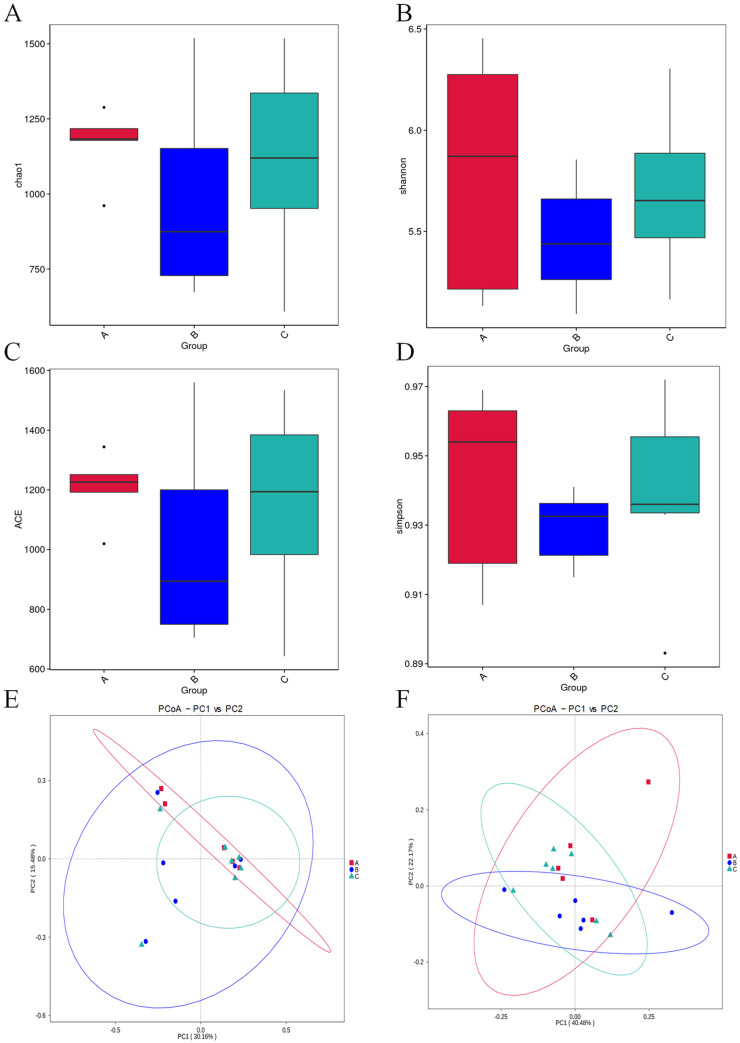
Microbial alpha and beta diversity analysis. (**A**) Chao1 index; (**B**) Shannon index; (**C**) ACE index; (**D**) Simpson index. The β diversity was reflected by the unweighted (**E**) and weighted (**F**) Unifrac PCoA analysis. The vertical columns and different colored spheres and dots represent different groups (A: music treatment group, B: white noise treatment group, C: control group).

The alpha diversity analysis index (chao1, Shannon, ACE, Simpson) of different groups under the 97% agreement threshold was calculated for every group and is shown in Figure 3A–D. Among them, Ga had the highest alpha diversity and Gb had the lowest; however, no statistical differences were observed for any of these four indexes. The unweighted (Figure 3E) and weighted (Figure 3F) Unifrac PCoA analysis to reflect β diversity showed no significant difference between the three groups, indicating a high overall similarity and low species diversity between these groups.

**Figure 4:**
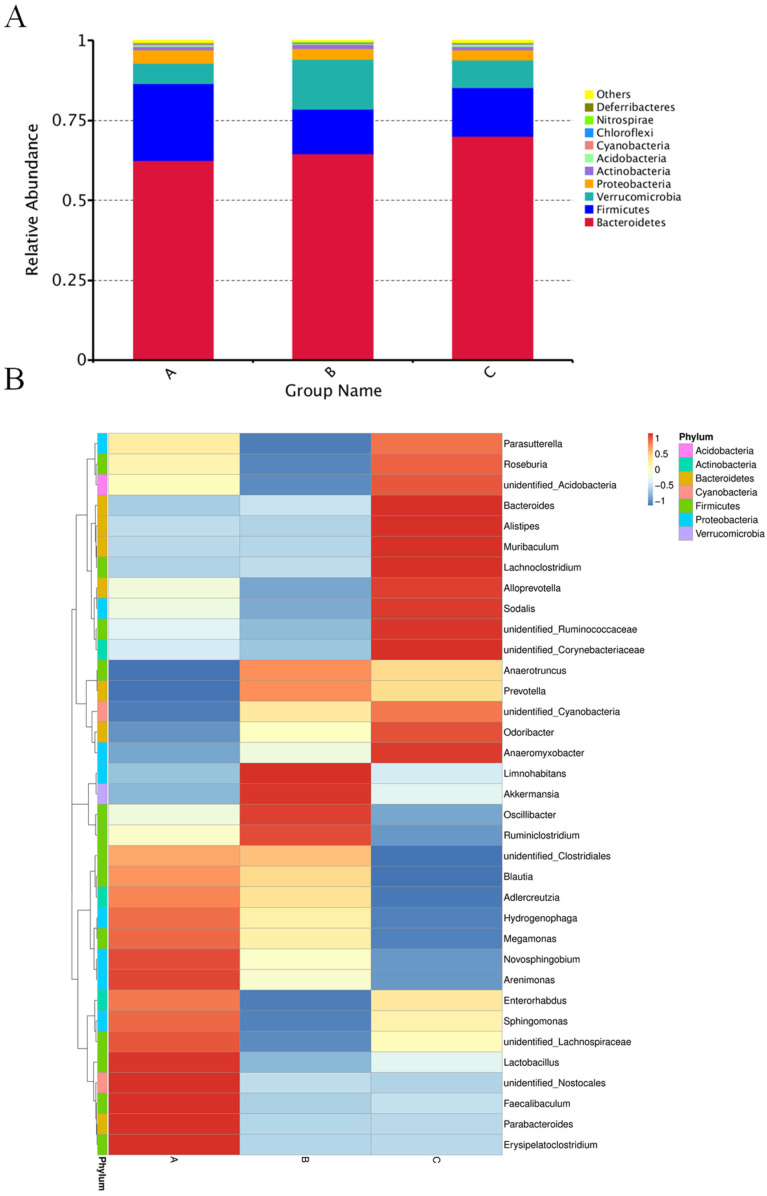
The bacterial composition’s relative abundance in different treatment groups at the phylum level (**A**) and the heat map of microbial distribution at the genus level. The color key scale shows the Z-score (**B**). The vertical columns represent different groups: (A) music treatment group, (B) white noise treatment group, (C) control group.

The relative abundance at the phylum levels is shown in Figure 4A. The distribution of bacteria at the phylum level in Ga was *Bacteroidetes* (62.60%), *Firmicutes* (23.95%), *Verrucomicrobia* (6.4%), *Proteobacteria* (4%), *Actinobacteria* (1%), *Acidobacteria* (0.4%), *Cyanobacteria* (0.3%), *Chloroflexi* (0.3%), *Nitrospirae* (0.1%), and *Deferribacteres* (0.02%). In Gb, the distribution of bacteria at the phylum level was *Bacteroidetes* (64.58%), *Firmicutes* (14.01%), *Verrucomicrobia* (15.63%), *Proteobacteria* (3.38%), *Actinobacteria* (1.1%), *Acidobacteria* (0.3%), *Cyanobacteria* (0.2%), *Chloroflexi* (0.1%), *Nitrospirae* (0.07%), and *Deferribacteres* (0.05%). In Gc, the distribution of bacteria at the phylum level was *Bacteroidetes* (70.03%), *Firmicutes* (15.30%), *Verrucomicrobia* (8.58%), *Proteobacteria* (3.27%), *Actinobacteria* (1.11%), *Acidobacteria* (0.529%), *Cyanobacteria* (0.15%), *Chloroflexi* (0.34%), *Nitrospirae* (0.14%), and *Deferribacteres* (0.04%). The *Firmicutes*/*Bacteroidetes* ratio for Ga, Gb, and Gc was 38.26%, 21.69%, and 21.84%, respectively. A more detailed distribution of bacteria was illustrated in the heat map at the genus level (Figure 4B). The three groups were differentiated by the different microbial abundance distribution. Ga was more abundant in unidentified *Clostridiales*, *Blautia*, *Adlercreutzia*, *Hydrogenophaga*, *Megamonas*, *Novosphingobium*, *Arenimonas*, *Enterorhabdus*, *Sphingomonas*, unidentified *Lachnospiraceae*, *Lactobacillus*, unidentified *Nostocales*, *Faecalibaculum*, *Parabacteroides*, and *Erysipelatoclostridium*. Gb was more abundant in *Limnohabitans*, *Akkermansia*, *Oscillibacter*, and *Ruminiclostridium*. Gc was more abundant in *Parasutterella*, *Roseburia*, unidentified *Acidobacteria*, *Bacteroides*, *Alistipes*, *Muribaculum*, *Lachnoclostridium*, *Alloprevotella*, *Sodalis*, unidentified *Ruminococcaceae*, and unidentified *Corynebacteriaceae*.

**Figure 5:**
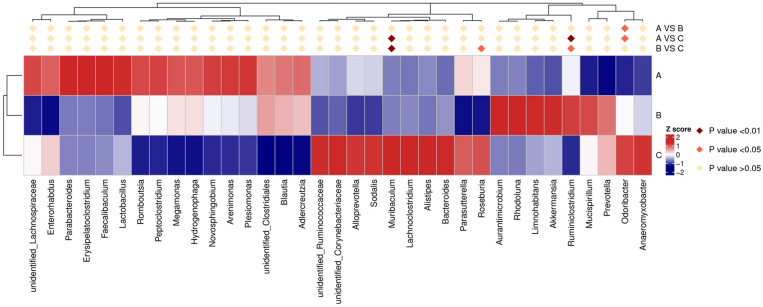
Metastats analysis of the samples at the genus level. The horizontal rows represent different groups: (A) music treatment group, (B) white noise treatment group, (C) control group. Vertical columns show the annotation information. The scale of the color key is based on the z-scores.

Furthermore, Metastats analyses were performed to identify statistically different bacteria at the genus level (Figure 5). The *Muribaculum* in Gc was significantly higher than Ga and Gb (*p* < 0.01), yet no significant difference was observed between Ga and Gb. The *Roseburia* in Gc was significantly higher than Gb (*p* < 0.05), but no significant difference was found between Ga and Gc or Gb. The *Ruminiclostridium* in Gc was significantly higher than Ga (*p* < 0.01) and Gb (*p* < 0.05), and no significant difference was observed between Ga and Gb. The *Odoribacter* in Ga was significantly higher than Gb (*p* < 0.05) and Gc (*p* < 0.05), but no significant difference was found between Gb and Gc.

### 3.6. Comparison of the Functional Pathway of the Microbiota

**Figure 6:**
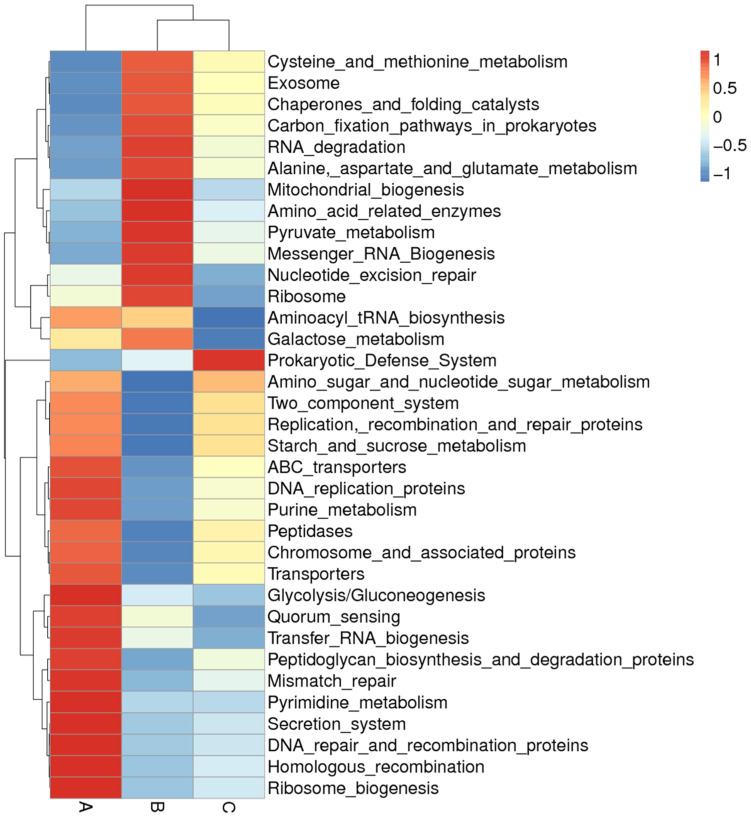
Level 3 functional prediction by Tax4Fun for the gut microbiota. The vertical columns represent different groups: (A) music treatment group, (B) white noise treatment group, (C) control group. Horizontal rows show predicted functions. The scale of the color key is based on the z-scores.

Tax4Fun was used for functional pathway prediction. Figure 6 shows the level 3 cluster heatmap for the predicted functions. The results show a distinctively different functional pattern between the three groups. The functional relative abundance in Ga was enriched in ABC transporters, DNA replication proteins, purine metabolism, peptidases, glycolysis/gluconeogenesis, quorum sensing, transfer RNA biogenesis, peptidoglycan biosynthesis and degradation proteins, pyrimidine metabolism, secretion system, etc. The functional relative abundance in Gb was eminent in cysteine and methionine metabolism, RNA degradation alanine, aspartate and glutamate metabolism, mitochondrial biogenesis, amino acid-related enzymes, pyruvate metabolism, etc. The functional relative abundance in Gc was enriched in the prokaryotic defense system for the level 3 prediction; however, level 2 of the functional prediction of Gc shows enrichment in the lipid metabolism and metabolism of terpenoids and polyketides (Figure S2). Interestingly, Figure S2 shows a distinguishable enrichment pattern for Ga, Gb, and Gc. Moreover, non-metabolism-related enrichment, such as elevated expression levels for cancers, the nervous system, drug resistance, and aging, were observed for the Gb group (Figure S2).

**Figure 7:**
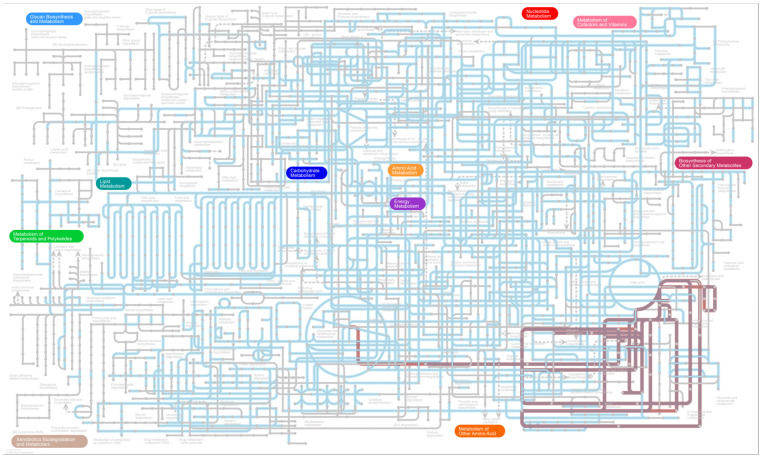
iPath3 illustration of the pathway difference between the intersection (blue) and complement (red) of the music treatment group, white noise treatment group, and control group.

The pathway differences between the intersection and the complement of Ga, Gb, and Gc were illustrated by iPath3 (Figure 7), and are shown in blue and red, respectively, in the complete landscape of the Kyoto Encyclopedia of Genes and Genome (KEGG) metabolic pathways. The sound treatments (Ga and Gb) were seen to affect amino acid metabolic pathways for D-glutamine and D-glutamate metabolism, biotin metabolism, and arginine and proline metabolism compared with the Gc group.

## 4. Discussion

The correlation between changes in the gut microbiota and the physiological status of mice following music or noise treatment was observed in this study, which supports our postulation that there exists a link between auditory stimuli (music or white noise), the gut microbiota, and the host’s physiology. Our findings reveal that exposure to Mozart’s K.448 composition increased the BW of the mice, while exposure to noise had the opposite effect. In addition, noise-induced stress led to an alteration in the mice’s gut microbiota by promoting the growth of specific bacterial species, whereas music exposure affected different types of gut microbes. The reconfiguration of gut microbiota not only modified the diversity and abundance of the gut microorganisms but also facilitated the alterations in immune responses and the expression of redox enzymes to cope with external stimuli. Simultaneously, whole-body metabolism underwent reprogramming to adapt to different pathway utilization. This research area is particularly intriguing for studying the potential interaction between sounds and the physical and psychological health of both humans and animals through the modulation of the gut microbiota. These results offer significant promise for a deeper understanding of the intricate mechanisms underlying the impact of music and noise exposure on human and animal well-being, mediated through changes in the gut microbiota.

It was found that the difference in the growth curve for Gc was higher than Gb and lower than Ga during the entire experiment period, indicating the white noise at 70–80 dB might hinder the weight gain of the mice. This is consistent with the previous findings in animal production [48,49,50]. Komkrit et al. compared the effect of light music and rock and roll on growing pigs and found that irregular and fast rhythm rock and roll music could lower the growth performance, while the light music group had a slightly higher performance in weight gain [50]. The white noise used in this study also had an irregular rhythm and is comparable with the rock and roll music in the finding. The more active behavior of the mice in the music and white noise treatment group is consistent with other studies of animal behavior [49,51].

Oxidative stress occurs when the balance between oxidation and antioxidant systems is disrupted. SOD and GSH-PX are two critical enzymes in antioxidant mechanisms. They are critical to controlling the accumulation of reactive oxygen species (ROS) and preventing oxidative stress [52]. MDA is usually the end product of lipid peroxidation, and serum LDH is an oxidative stress marker. The slopes of the weight growth curves for the three groups were identical before the third week, but the slope for the growth curve of Gb decreased after the third week. This phenomenon might be the result of the disturbance of animal physiological homeostasis through the depression of the antioxidant system with the white noise-induced accumulation of oxidative stresses. This hypothesis was substantiated by the lower serum SOD and GSH-PX values, and higher LDH and MDA values in Gb, indicating the suppression of antioxidant activity and the accumulation of oxidative damage. These results are in line with the previous studies that linked noise treatment with stress occurrence in animal production [7,48].

Sound treatment could influence the activity of the T lymphocytes and promote the secretion of cytokines, such as IL-1, IL-2, and IFN-γ, thereby affecting the animals’ immune systems and animal welfare [53,54]. IFN-γ and IL-1β are two important indicators for the status of the immune system. The relative expression of IFN-γ and IL-1β were lower in Gb, which indicates a possible suppression effect of noise on the immune system. Other recent reports have found a similar effect of noise on the immune system [55]. Ga had similar IFN-γ and IL-1β relative expression levels compared with Gc, indicating that classical soothing music had no side effects on the animals. Previous studies on the effect of light music have also come to a similar result [2].

Gut microbiota (GM) in animal and human intestines have a profound influence on maintaining their health and the formation of diseases [56]. The intricate interaction between the environment, host, and gut microbiota as a reciprocal relationship through co-evolution remains to be explored [57]. Sound as an environmental factor has been studied for its relationship with the physiological responses of hosts (human or animal) and the relevant gut microbiota [30,58,59]. In this study, the α-diversity parameters in Gb were lower than Ga and Gc, yet the parameters in Ga were slightly higher than Gc. This shows that the sound could have an impact on the composition of GM. The β diversity showed a large overlap between each group, indicating that the sound treatment could influence the composition of GM, but was not intensive enough to change the entire landscape of it. Niu et al. also found this phenomenon in a study on the effect of music interference while feeding mice [60].

The phylum-level relative abundance of GM in Ga, Gb, and Gc revealed that *Bacteroidetes*, *Firmicutes*, *Verrucomicrobia*, and *Proteobacteria* were dominant among all the groups. Compared with Gc, the proportion of *Firmicutes* increased in Ga but decreased in Gb, and the proportion of *Verrucomicrobia* decreased in Ga but increased in Gb. Swapping *Firmicutes* for *Verrucomicrobia* might be a coping mechanism for mice in the white noise environment. Cui et al. also reported a decrease in the relative abundance of *Firmicutes* and an increase in *proteobacteria* in the GM composition of rats exposed to chronic noise. *Verrucomicrobia* is not in the dominant bacteria list of the study [31]. This implies that the response of different compositions of GM has a different response pattern to noise depending on the dominant bacteria, and *Firmicutes* is important in this process since the concurrence of change in the relative abundance of *Firmicutes* and the noise treatment have been observed in many studies [31,48,59,60]. Gut *Firmicutes* could influence host nutrition and metabolism through SCFA synthesis [61] and could interact with the intestinal mucosa, thereby contributing to gastrointestinal homeostasis [62]. The *Firmicutes*/*Bacteroidetes* ratio has been calculated as a biomarker for many diseases [63]. Jeongshin et al. found that the *Firmicutes*/*Bacteroidetes* ratio for patients with breast cancer was three times lower than in healthy controls [64]. Koliada et al. reported that an increase in the *Firmicutes*/*Bacteroidetes* ratio was significantly associated with increasing BMI in an unadjusted logistic regression model (OR = 1.23, 95% CI 1.09–1.38) [65]. The *Firmicutes*/*Bacteroidetes* ratio in Ga was almost two times that of Gb and Gc, indicating the classical music treatment could increase the *Firmicutes*/*Bacteroidetes* ratio in GM. The subsequent heatmap analysis at the genus level showed Ga was more abundant in *Lactobacillus*, *Faecalibaculum*, *Megamonas*, etc. These three genera are contained in the *Firmicutes* phylum, which contains many beneficial bacteria [65]. *Lactobacillus* and *Faecalibaculum* have been reported to produce short-chain fatty acids and antibacterial substances to prevent pathogens from affecting the gut homeostasis of the host [66,67]. From the discussion above, one may conclude that classical music could have a beneficial effect on mice by increasing the abundance of probiotics that reside in the phylum of *Firmicutes*; however, white noise had an adverse effect on the mice by decreasing the abundance of *Firmicutes*. Still, this is only preliminary speculation of the complete and intricate function world of GM.

A functional analysis of GM revealed that purine metabolism and quorum sensing (QS) are more abundant in Ga. QS is a complex network of communication between bacteria that is mediated by autoinducers (AIs). Through AIs, bacteria could influence one another and participate in the crosstalk between the host and parasitic microbes to balance their population density and gene expression for the benefit of the microbial community and the host [68,69]. Purine metabolites have been reported to be involved in purine signaling in hosts as signaling messengers [70]. The cysteine and methionine metabolism are more abundant in Gb. Methionine is important in one-carbon metabolism and is involved in cysteine synthesis in gut microbiota [71]. One-carbon metabolism provides building blocks for the biosynthesis of DNA, proteins, and lipids [72]. It was further found that non-metabolism-related gene expression appeared for cancers, the nervous system, drug resistance, aging, and oxidative stresses for white noise exposure, which coincides with many previous studies [58,73,74].

The lipid metabolism and metabolism of terpenoids and polyketides were more abundant in Gc. The iPath analysis showed the sound treatment (Ga + Gb) influenced a specific portion of metabolism in the KEGG metabolic pathway. The discussion above indicates the music and the white noise influenced the metabolism in the microbial community. Considering that the functional analysis was based on 16s rRNA gene sequencing data by targeting relatively small variable regions, the prediction for the function and metabolism might be inaccurate; further studies with metagenomic sequencing analysis would be beneficial to reveal more detailed and precise results.

This work delves into three crucial facets of farm animal welfare research: music, noise, and microbiota. In the established conceptual framework for farm animal welfare, music has been employed to mitigate unfavorable sounds or provide sensory stimulation [75,76]. Noise, on the other hand, is undesirable and is addressed by improving housing facility design or masking it with music [77,78]. The gut microbiota is targeted for intervention with probiotics to enhance animal behavior and immunity, particularly through the microbiota–gut–brain axis [79,80]. The simultaneous changes observed in the mice’s microbiota and their physiological status following music or noise treatments suggest a potential link. This connection hints at the possibility of further exploring the beneficial effect of music, and the detrimental effect of noise on farm animals could be further explored through the microbiota–gut–brain axis. The data for the negative effect of noise on animal microbiota and physiology observed in this study could also support the initiative of the UN Convention on the Law of the Sea (UNCLOS) to require that the noise impact on marine animals be evaluated for future marine resources exploitation, as well as the technical guidance of the Convention on the Conservation of Migratory Species of Wild Animals (CMS) on shipping, seismic airgun surveys (for oil and gas exploration), and pile driving (for offshore wind farms and other marine infrastructure), which are three major sources of marine noise pollution affecting the welfare of marine animals.

Although the music and noise impacts on the animal model were not substantial, they seemed to be observable. Bearing in mind the direct effects of music on the gut microbiota and the underlying mechanisms that are still not well understood, more research is needed to elucidate the specific pathways through which music and noise might influence the gut microbiota and to determine the extent of these effects.

## 5. Conclusions

This study set out to investigate the possible relationship between music and white noise, gut microbiota, and the physiological status of mice. The results show that white noise has a negative influence on the physiological status and microbiota of mice, characterized by decreased antioxidant enzyme activity, increased lipid peroxidation, lowered relative gene expression of immune-related genes, and a decreased proportion of Firmicutes in the microbial composition. On the contrary, music has a beneficial effect on mice, supported by the increase in the weights of the mice and increased ratio of *Firmicutes*, a phylum that is generally considered beneficial to the host, in the microbiota. These findings could serve as evidence to support music application in animal production to reduce stress and improve animal welfare, and measurements to reduce the noise produced by animal operation facilities are also pivotal points for maintaining animal health and improving animal performance. Although this study contributes to understanding the relationship between music, white noise, and the gut microbiota, the subtle mechanism under the concurrence of the sound treatment, the change in the gut microbiota, and the host physiology status are still understudied. Further studies on livestock and the underlying mechanism are needed to consolidate the results.

## Data Availability

The sequencing data of this study have been uploaded to the Genome Sequence Archive in BIG Data Center (https://ngdc.cncb.ac.cn/?lang=en, accessed on 8 May 2021), Beijing Institute of Genomics (BIG), Chinese Academy of Sciences, with the accession number PRJCA018758.

## Supplementary Materials

The following supporting information can be downloaded at: https://www.mdpi.com/article/10.3390/microorganisms11092272/s1, Figure S1: Excerpt of the music notes of the sonata for two pianos in D major, K.448 for the music treatment group (A). The illustration of white noise signals (B) and histogram of white noise following a gaussian distribution for the white noise treatment group (C).; Figure S2: Level 2 functional prediction by Tax4Fun for the gut microbiota. The vertical columns represent different groups (A: music treatment group, B: white noise treatment group, C: control group). Horizontal rows show predicted functions. The scale of the color key is based on the z-scores.; Table S1: amplicon sequencing results for every sample; Table S2: Relevant information of primers used in Quantitative real-time PCR.
