# Supplementary material for: Effects of Music and White Noise Exposure on the Gut Microbiota, Oxidative Stress, and Immune-Related Gene Expression of Mice
Source: Microorganisms. 2023 Sep 10;11(9):2272. doi: 10.3390/microorganisms11092272 (PMC10536120; doi:10.3390/microorganisms11092272)
Supplement: Supplementary file 1 [file microorganisms-11-02272-s001.zip › microorganisms-2566911-supplementary.pdf]

Table S1. amplicon sequencing results for every samples.

Sample Name	Raw reads	Clean reads	Effective reads	AvgLen(nt)
A1	81,023	79,136	55,234	421
A2	94,784	92,972	67,880	420
A3	92,577	90,896	65,642	422
A4	98,468	95,996	65,448	421
A5	85,247	82,860	64,337	424
B1	79,450	78,051	64,311	420
B2	91,562	89,711	68,906	419
B3	85,277	83,618	63,802	419
B4	85,458	83,407	68,308	423
B5	98,734	97,116	69,445	420
B6	61,323	59,890	52,061	418
C1	79,816	77,899	61,255	422
C2	94,902	92,943	63,548	421
C3	88,657	86,499	68,072	418
C4	84,125	81,853	62,264	421
C5	92,519	90,397	65,719	422
C6	91,447	89,470	61,990	420
C7	80,247	78,492	67,647	423

Table S2. Relevant information of primers used in Quantitative real-time PCR

Gene name	Accession numbers	Primers	Sequence(5'-3')	Product size (bp)	Anneal temperature (°C)
GAPDH	NM_008084.2	M-GAPDH-S	CCTCGTCCCGTAGACAAAATG	133	60
		M-GAPDH-A	TGAGGTCAATGAAGGGGTCGT		60
IFN-γ	NM_008337.4	M-IFNG(rz）-S	CTCAAGTGGCATAGATGTGGAAG	251	60
		M-IFNG(rz）-A	TGACCTCAAACTTGGCAATACTC		60
IL-1β	NM_008361.4	M-il1b(RZ)-S	GCATCCAGCTTCAAATCTCGC	256	60
		M-il1b(RZ)-A	TGTTCATCTCGGAGCCTGTAGTG		60

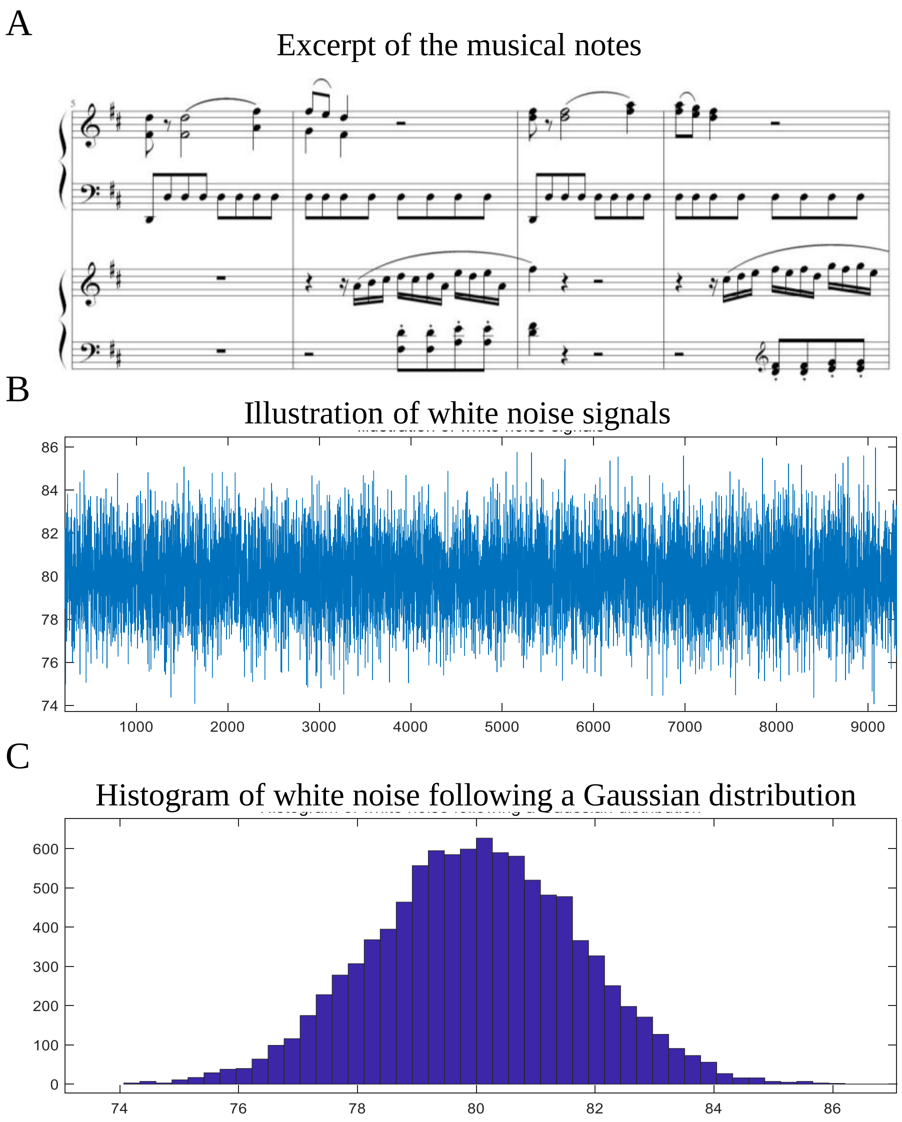

Figure S1. Excerpt of the music notes of the sonata for two pianos in D major, K.448 for the music treatment group (A). The illustration of white noise signals (B) and histogram of white noise following a gaussian distribution for the white noise treatment group (C).

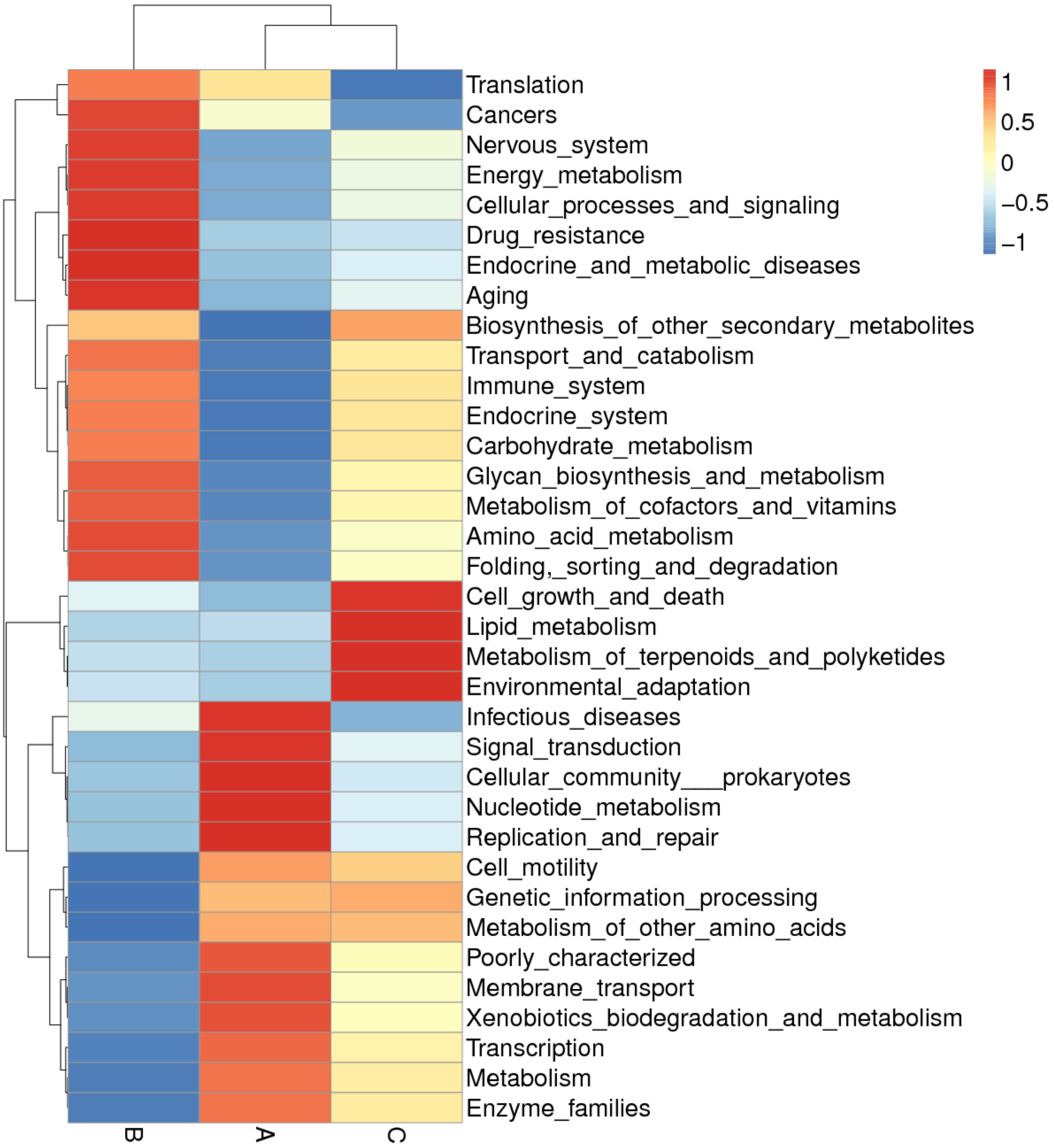

Figure S2. Level 2 functional prediction by tax4fun for the gut microbiota. The vertical columns represent different groups (A: music treatment group, B: white noise treatment group, C: control group). Horizontal rows show predicted functions. The scale of the color key is based on the z-scores.
